# The impact of Undetectable=Untransmittable and viral suppression on condomless sex among mixed HIV-status couples in Canada

**DOI:** 10.1371/journal.pone.0332926

**Published:** 2025-10-09

**Authors:** Min Xi, Sandra Bullock, Joshua B. Mendelsohn, Veronika Moravan, Ann N. Burchell, Darrell H. S. Tan, Bertrand Lebouché, Jean-Pierre Routy, Amrita Daftary, Tamara Thompson, Liviana Calzavara

**Affiliations:** 1 Department of Public Health Sciences, Dalla Lana School of Public Health, University of Toronto, Toronto, Ontario, Canada; 2 Toronto General Hospital Research Institute, University Health Network, Toronto, Ontario, Canada; 3 ICES Central, Toronto, Ontario, Canada; 4 College of Health Professions, Pace University, New York, New York, United States of America; 5 VM Stats, Toronto, Ontario, Canada; 6 Department of Family and Community Medicine, MAP Centre for Urban Health Solutions, Li Ka Shing Knowledge Institute, St. Michael’s Hospital, Unity Health Toronto, Ontario, Canada; 7 Division of Infectious Diseases, St. Michael’s Hospital, Unity Health Toronto, Ontario, Canada; 8 Department of Medicine, University of Toronto Temerty Faculty of Medicine, Toronto, Ontario, Canada; 9 Institute of Medical Science, University of Toronto, Toronto, Ontario, Canada; 10 MAP Centre for Urban Health Solutions, St. Michaels’ Hospital, Unity Health Toronto, Toronto, Ontario, Canada; 11 Centre for Outcomes Research and Evaluation, Research Institute of the McGill University Health Centre, Montreal, Quebec, Canada; 12 Department of Family Medicine, McGill University, Montreal, Quebec, Canada; 13 Chronic Viral Illness Service, Royal Victoria Hospital, McGill University Health Centre, Montreal, Quebec, Canada; 14 Chronic Viral Illness Service and Division of Hematology, McGill University Health Centre, Montreal, Quebec, Canada; 15 Research Institute of the McGill University Health Centre, Montreal, Quebec, Canada; 16 School of Global Health, and Dahdaleh Institute of Global Health Research, York University, Toronto, Ontario, Canada; 17 Faculty of Health Sciences, Douglas College, Coquitlam, British Columbia, Canada; University of Ghana College of Health Sciences, GHANA

## Abstract

**Introduction/Objectives:**

Undetectable = Untransmittable (U = U) means that virally suppressed HIV-positive partners cannot transmit HIV. With advancements in HIV care and the increasing number of mixed HIV-status relationships, we quantified U = U agreement (agreeing/disagreeing that U = U is true) among people in mixed HIV-status relationships, assessed response concordance between partners, quantified the association between dyad-level U = U agreement and condomless sex, and estimated interaction effects of viral suppression.

**Methods:**

A cross-sectional opportunistic and snowball sampled survey explored condom use, U = U agreement, viral suppression, sociodemographic/relationship characteristics, and sexual behavior among 306 participants (153 matched dyads) in mixed HIV-status relationships (2016–2018). Bivariate analyses identified sociodemographic/relationship characteristics associated with U = U agreement. Cohen’s Kappa assessed response concordance between partners. Firth logistic regression estimated associations between dyad-level U = U agreement and condomless sex with effect modification by viral suppression.

**Results:**

Of 293 responses to the U = U question, 53.2% agreed with U = U. Agreement was associated with younger age (p = 0.006) and shorter duration of HIV in relationships (p = 0.034).

Concordance between partners was higher for factual questions (kappa>0.50) and lower for belief/decision-making questions (kappa<0.40). The predicted probability of always having condomless sex was 0.60 (95% CI:0.57,0.64) when dyads agreed with U = U and the HIV-positive partner was virally suppressed. The likelihood of always having condomless sex was low when the HIV-positive partner was not virally suppressed, whether or not dyads agreed with U = U (predicted probability range: 0.08;95% CI:0.06,0.11 to 0.25;95% CI:0.17,0.33), or when only one partner agreed with U = U (predicted probability range:0.02;95% CI:0.01,0.02 to 0.11;95% CI:0.07,0.15).

**Conclusions:**

Dyad-level U = U agreement, viral suppression, and views of both partners were key factors in mixed HIV-status couples’ decisions to have condomless sex. Future representative studies among sexual identity and racialized minority sub-populations are needed to better understand how mixed HIV-status relationships receive and apply U = U messaging, with a focus on partner age and the duration of HIV within relationships.

## Introduction

Advancements in HIV care have improved health and increased life expectancy among people living with HIV [1–3]. In the United States, approximately 25% of heterosexual people living with HIV have a primary sexual partner who is HIV-negative [4–12]. Data from Uganda, Zambia, and Rwanda suggested that 50–70% of people living with HIV are involved in a mixed HIV-status relationship [13,14]. The risk of HIV transmission within such relationships has been shown to range between 0 and 20% per annum, depending on sexual behavior and prevention methods used (e.g., use of antiretroviral therapy [ART], condoms, and/or HIV pre-exposure prophylaxis) [15]. Prevention of HIV transmission remains a concern for people in mixed HIV-status relationships [16]. Adherence to ART and consistent condom use continue to constitute two key HIV prevention methods for mixed HIV-status partners [17].

Since 2016, strong empirical data have shown that people living with HIV who are on ART and achieve a sustained undetectable viral load (i.e., viral suppression) cannot sexually transmit the virus [18–21]. This concept is referred to as “Undetectable=Untransmittable” (U = U) [18–21]. U = U has been endorsed by prominent public health agencies, HIV experts, and AIDS Service Organizations [22]. As of December 2023, U = U has been endorsed by over 1099 organizations from 105 countries [22]. As a key component of U = U, the viral suppression status of an HIV-positive partner may play a key role in a mixed HIV-status couple’s decision to use condoms during sex.

In Canada and 68 other countries, people living with HIV are legally required to disclose their HIV status to their sexual partners in cases of “significant risk of serious bodily harm,” regardless of whether HIV transmission had occurred [23,24]. By reducing HIV-related stigma [25], U = U messaging is poised to help reduce the overcriminalization of HIV [26]. In doing so, the benefits of agreement with U = U (i.e., understanding/believing U = U to be true) may help improve HIV testing rates and linkage to care [25] while enhancing sexual pleasure for people in mixed HIV-status relationships through HIV-protected, condomless sex [27]. The perceived lower risk of HIV transmission may impact decisions taken by partners in mixed HIV-status relationships regarding condom use [18]. Many healthcare professionals continue to recommend consistent condom use to ensure optimal protection against HIV transmission [28]. People may continue to use condoms to protect against other sexually transmitted infections and unwanted pregnancy [28].

While condom use remains an important method for reducing the risk of HIV transmission, the proportion of mixed HIV-status couples who use condoms varies from 20% to 75% across studies [9,29–32]. Several factors have been associated with increased condom use during sexual intercourse among mixed HIV-status couples including increased age [33], more than 11 years of education [34], greater than $10,000 annual income [35], self-identifying as a woman [16,36,37], self-identifying as black [38], sexual behavior (e.g., multiple sexual partners [34], frequency of sexual intercourse [33], desire for sexual pleasure [39]), and relationship factors [16,35]. These sociodemographic, sexual behavior, and relationship factors have been shown to be associated with U = U agreement. For example, awareness and agreement with U = U were found to be lower among sexually active adults who self-identify as black and heterosexual compared to other gender and sexual minorities (e.g., men who have sex with men) [40–43] and among HIV-negative compared to HIV-positive people [44]. Younger age, higher education, higher income, increased HIV knowledge, and having many sexual partners, have all been found to be associated with increased agreement with U = U [46,47]. Notably, few studies that described participants’ agreement with U = U have captured the opinions of both partners in a mixed HIV-status relationship. Dyad-level data are crucial given that the views of each partner in a relationship can impact health decisions and sexual behaviors (e.g., condom use) of both partners in the relationship [47].

To date, however, there have been few studies that have investigated the impact of agreement with U = U on condom use among people in mixed HIV-status relationships. One mixed methods study [16] and one qualitative study [48] asked both partners in a heterosexual mixed HIV-status relationship [16], healthcare providers [48], and HIV-negative people in mixed HIV-status relationships [48], broad questions regarding participants’ agreement with U = U and their willingness to use HIV prevention interventions including condoms and HIV pre-exposure prophylaxis. Findings were mixed on the impact of agreement with U = U and viral suppression on the perceived need and use of other HIV prevention interventions [16,48]. As these studies were exploratory in nature and involved small samples, they were not able to quantify the relationship between agreement with U = U and condom use [16,48]. Such information could help inform future educational campaigns to improve HIV knowledge, prevent HIV transmission, and reduce HIV-related stigma.

In response to this evidence gap, we sought to: (1) quantify the proportion of individuals and dyads involved in mixed HIV-status relationships in Canada who agreed with U = U; (2) describe dyad-level concordance in agreement with U = U and other relationship and sexual behavior-related characteristics; (3) quantify the association between dyad-level agreement with U = U and condomless sex; and (4) estimate the effect of viral suppression as a potential effect modifier of the association between dyad-level U = U agreement and condomless sex. We hypothesized that condomless sex would be more likely if both partners agreed with U = U and the HIV-positive partner was virally suppressed (i.e., when the concept of U = U could be applied) compared with a null scenario where neither partner agreed with U = U or where the HIV-positive partner was not virally suppressed (i.e., when the concept of U = U would not be immediately relevant).

## Materials and methods

We analyzed 306 of 613 total participants who completed the survey component of the Positive Plus One (PP1) study who were part of a matched dyad in a current mixed HIV-status relationship. Eligibility criteria, recruitment strategy, and processes of the PP1 study have been described in detail elsewhere [49]. PP1 was a Canadian nation-wide study that aimed to understand sociodemographic characteristics, relationship satisfaction, HIV transmission risk, perceived needs, access to supportive services, and subjective experiences of people in mixed HIV-status relationships.

### Recruitment

PP1 used a multipronged recruitment strategy to maximize the number of participating HIV-positive and HIV-negative partners [49]. Recruitment efforts took place between January 1, 2016, and June 30, 2018. Due to the lack of an existing sampling frame, opportunistic sampling was used to recruit participants at non-governmental organizations, AIDS service organizations, medical clinics, and community pharmacies using word of mouth and social media, television, radio, and newspaper media advertising across Canada (University of Toronto-associated sites: January 1, 2016-June 30, 2018; Toronto Public Health-associated sites: April 16, 2016-June 30, 2018; McGill University-associated sites: May 25, 2016-June 30, 2018; University of Saskatchewan-associated sites: June 18, 2016-June 30, 2018; Saskatchewan Health Region-associated sites: October 7, 2016-June 30, 2018; Regina Qu’Appelle Health Region-associated sites: October 7, 2016-June 30, 2018; Prince Albert Parkland Health Region-associated sites: January 5, 2017-June 30, 2018; St Michael’s Hospital-associated sites: January 9, 2017-June 30, 2018; and Nova Scotia Health Authority-associated sites: July 17, 2017-June 30, 2018). Participants received a link to the survey to pass the study information to their partners and others they know who may be involved in mixed HIV-status relationships. Although no quotas were set, we made deliberate efforts to recruit a diverse sample across regions, genders, and sexual identities.

Participants completed eligibility screening, provided consent, and filled out surveys either through the study website or by phone with assistance from a research team member. All participants indicated their written informed consent in the online consent form following eligibility screening. Participants in each dyad were surveyed separately. Consent of both partners within a mixed HIV-status relationship was required to link their responses.

### Participants

PP1 sought to recruit adults (≥18 years) who: (1) were in a current or past (i.e., within two years prior to study enrolment) mixed HIV-status relationship; (2) had knowledge that their relationship was of mixed HIV-status status; (3) lived in Canada at the time of the survey and during at least part of the relationship; and (4) could speak and/or read and write English or French. For this study, a mixed HIV-status relationship was defined as a primary relationship where one partner was HIV-positive and the other was HIV-negative. To be considered a primary relationship, the index partner (first partner enrolled in the study) had to consider their relationship as “dating,” “together,” or “a couple.” This definition was used to distinguish and screen out people in casual and sex-only relationships. There were no restrictions on the sex and gender of partners involved in a mixed HIV-status relationship.

For people in a current mixed HIV-status relationship, the index partner (first partner of a couple to enroll in the study regardless of whether they were HIV-positive or HIV-negative) was encouraged to invite their partner to the study. For polyamorous relationships, the index partner could invite one mixed HIV-status partner to be matched for analysis; other partner(s) could join, but were not matched for analysis.

Of 613 people recruited into the study, 355 (58%) were HIV-positive, 258 (42%) were HIV-negative, 540 (88%) were in current, and 73 (12%) had been in past relationships. Of the 387 index partners in current relationships, 297 (77%) invited their partner to enroll, and 153 (39%) of those ultimately enrolled. The present analysis only includes participants (n = 306) who were in one of the 153 matched dyads.

### Measures

A structured survey, evaluated at a grade eight level or less, was administered separately to each enrolled partner in English or French to collect data on a broad range of topics relevant to mixed HIV-status relationships. Complex terms within the questionnaire were hyperlinked to lay definitions to provide clarity. Dyad-level variables were created for each mixed HIV-status relationship by combining each partner’s individual responses.

#### Condomless sex.

The dependent variable was dyad-level condom use with a mixed-HIV status primary partner over the three months prior to survey completion. This variable was created using each partner’s individual responses to time since last sex, frequency of sex, and frequency of condom use during sex in the three months prior to survey completion. If a participant did not indicate when they first and last had sex (how many years/months ago) or indicated that they did not know the answer, condom use questions were skipped by the online survey. When only one partner responded to questions on sexual intercourse, their response was imputed as the dyad-level response. When partners’ responses were discordant, the dyad-level variable was set to default to the lowest level of condom use reported by either partner as this best reflected HIV risk and potential self-report bias. Response options for frequency of condom use during sex in the three months prior to survey completion included: “never use condoms”, “sometimes use condoms,” and “always use condoms.” Discordance in responses to the frequency of condom use during sex between partners was almost always between “sometimes use condoms” and either “never use condoms” or “always use condoms” (S1 Table).

Those who reported last having sex more than three months prior to survey completion were considered to have no sexual activity in the past three months. Those involved in male-male or male-female relationships who did not partake in anal and/or vaginal sex were considered to have no penetrative sex in the past three months. For couples who were not able to participate in penetrative intercourse, condom use questions were marked as not applicable.

#### Agreement with U=U.

The primary independent variable of interest was agreement with the following U = U statement: “When a person’s viral load is undetectable, they can safely have intercourse with their partner without a condom.” Each participant reported their level of agreement with the statement on a 5-point Likert scale (1=”strongly disagree” and 5 = ”strongly agree,” with response options for “not applicable” and “don’t know”). For analysis, we defined agreement with the U = U statement within the dyad, which we refer to as “dyad-level agreement.” Dyad-level agreement with the U = U statement was considered concordant if both partners agreed with the statement (i.e., agree or strongly agree) or both partners disagreed/were neutral regarding the statement (i.e., disagree, strongly disagree, or neutral). For regression analyses, dyad-level agreement was categorized as (1) both partners agreeing with the statement (i.e., both partners agreed or one partner agreed and one partner was neutral); (2) mutual disagreement or neutrality towards the statement (i.e., both partners disagreed/were neutral or one partner disagreed and one neutral); or (3) opposing views (i.e., one partner agreed and one partner disagreed).

#### Viral suppression.

Both partners were asked whether the HIV-positive partner’s viral load was suppressed at the time of the survey. Where partners disagreed, the HIV-positive partner’s response to the question was coded as the dyad-level variable.

#### Covariates.

Survey participants self-reported their age, identities (gender, sexual, and ethnic), socioeconomic status (education, employment status, and income), and region (province, city/town size). They provided information on the HIV-positive partner’s use of ART and relationship characteristics such as relationship duration, timing of HIV diagnosis in the relationship, sexual behavior (frequency of sexual intercourse, condom use, involvement in concurrent relationships), desire for children, sexual satisfaction, and overall relationship satisfaction.

### Statistical analyses

Descriptive statistics were generated for participant-level and dyad-level responses. Descriptive statistics for participant-level responses were generated for all participants and stratified by HIV status. Differences between partners for participant-level responses were tested using chi-square or Fisher’s exact test for categorical variables, Wilcoxon signed-rank test for ordinal variables, and paired t-test for continuous variables.

Two hundred and ninety three of the 306 participants (95.8%) responded to questions on agreement with U = U. The other 13 respondents responded with “don’t know” or missed the question. For the 293 participants who responded to the question on agreement with U = U, agreement with U = U by individual participant and dyad characteristics was described using frequencies and percentages for categorical variables and means and standard deviations for continuous variables. The association between participant- and dyad-level characteristics and agreement with U = U was analyzed using the chi-square test or Fisher’s exact test for categorical variables, Wilcoxon rank-sum test for ordinal variables, and t-test or analysis of variance (ANOVA) for continuous variables. Tukey’s honestly significant difference test was used for ANOVA post-hoc testing.

Survey responses were tested for concordance between partners in a dyad. Concordance was presented as a frequency and percentage by using unweighted Cohen’s Kappa and quadratic weights for nominal and ordinal variables, respectively. A kappa value of 0.41–0.60 was considered as moderate concordance, 0.61–0.80 was substantial concordance, and 0.81–1.00 was near-perfect concordance [50]. The direction of discordance within dyads was tested with McNemar’s test or McNemar-Bowker test (categorical variables), Wilcoxon signed-rank test (ordinal variables), and Bland-Altman limits of assessment and bias test (continuous variables). The Bland-Altman 95% limits of agreement are shown in the same units as the original variable along with estimated bias with a 95% confidence interval [51]. The differences were calculated as the HIV-positive partner’s response minus the HIV-negative partner’s response.

Bivariate and multivariable modeling of associations between dyad-level agreement with U = U and always having condomless sex was conducted using Firth logistic regression to obtain crude and adjusted odds ratios (OR and aOR), and confidence intervals (CI). Firth logistic regression enhanced model fit, which is crucial when working with small datasets [52]. Relationship duration and sexual agreement between partners were not entered into the model as they were strongly correlated with age and invovlement in concurrent relationships, respectively. Attempting to conceive a child with a partner was not entered into the model as it led to non-convergence of the model due to zero cell counts. The role of viral suppression as a potential effect modifier in the relationship between dyad-level agreement with U = U and always having condomless sex was examined using an interaction term in multivariable logistic regression models. Where there was strong evidence for an interaction effect, stratified effects were calculated using the “multicomp” package in R to examine group differences. Given the small sample size, we used backward elimination to increase statistical power by reducing the number of covariates in our model. We removed the covariate with the highest penalized Likelihood Ratio Test p-value at each step, until only predetermined covariates (relationship type, age of the HIV-positive partner, and age difference between partners) and covariates with p-value<0.05 remained.

All analyses were conducted using R software version 4.2.2 (R Foundation for Statistical Computing, Vienna, Austria). All p-values were two-sided and statistical significance was determined using p-value<0.05.

### Ethics

This study received ethics approval from the University of Toronto research ethics board (REB) (Protocol 31855). The study also underwent review and obtained approval from REBs at McGill University (2017–1779, 16–035-MUHC, eReviews_5368), University of Saskatchewan (15–399), St. Michael’s Hospital (16–343), Toronto Public Health (2016-02), Nova Scotia Health Authority (NSHA REB ROMEO FILE #: 1022121), Prince Albert Parkland Health Region (no REB number), and Regina Qu’Appelle Health Region (REB-15–133).

## Results

### Participant- and dyad-level characteristics

Three hundred and six participants were recruited; no difference was identified by region and period of recruitment (Kruskal-Wallis p-value: 0.962; data not shown). Participant- and dyad-level characteristics of the 306 participants (153 dyads) are presented in Tables 1 and 2, respectively. The mean age of the sample was 43 years. Half self-identified as gay men (51.6%) or were involved in a same-sex relationship between men (56.2%). Most participants (70.5%) self-identified as white. Few differences were observed between HIV-positive and HIV-negative partners except that a larger proportion of HIV-negative partners self-identified as heterosexual men (27.5% versus 9.8%; p < 0.001) and were involved in concurrent relationships (63.4% versus 51.3%; p = 0.044) compared to HIV-positive partners.

**Table 1:** Characteristics of individual study partners in current mixed-HIV status relationships (N = 306).

Individual Characteristics	Total (N = 306)	HIV-positive partner (n = 153)	HIV-negative partner (n = 153)	p-value^a^
Gender identity Woman Man All other	65 (21.2%) 234 (76.5%) 7 (2.3%)	42 (27.5%) 106 (69.3%) 5 (3.3%)	23 (15.0%) 128 (83.7%) 2 (1.3%)	**0.010**
Sexual identity Heterosexual Gay Bisexual All other	114 (37.3%) 163 (53.3%) 19 (6.2%) 10 (3.2%)	53 (34.6%) 82 (53.6%) 12 (7.8%) 6 (4.0%)	61 (39.9%) 81 (53.0%) 7 (4.5%) 4 (2.6%)	0.515
Gender * Sexual identity Gay man Heterosexual women Heterosexual man All other	158 (51.6%) 55 (18.0%) 57 (18.6%) 36 (11.8%)	79 (51.6%) 36 (23.5%) 15 (9.8%) 23 (15.0%)	79 (51.6%) 19 (12.4%) 42 (27.5%) 13 (8.5%)	**<0.001**
Age *Mean (SD)*	43.2 (11.7)	43.4 (11.2)	43.0 (12.2)	0.754
Racial identity White Black Hispanic Aboriginal All other Missing	213 (70.5%) 24 (7.9%) 18 (6.0%) 17 (5.6%) 30 (9.9%) 4	106 (70.7%) 9 (6.0%) 11 (7.3%) 9 (6.0%) 15 (10.0%) 3	107 (70.4%) 15 (9.9%) 7 (4.6%) 8 (5.3%) 15 (9.9%) 1	0.656
Education Less than secondary school diploma Secondary school diploma Beyond secondary school Missing	29 (9.5%) 60 (19.7%) 216 (70.8%) 1	17 (11.2%) 30 (19.7%) 105 (69.1%) 1	12 (7.8%) 30 (19.6%) 111 (72.5%) 0	0.437
Personal annual income Less than $20,000 $20,000 – $49,999 $50,000 or more Missing	91 (30.1%) 109 (36.1%) 102 (33.8%) 4	51 (34.0%) 56 (37.3%) 43 (28.7%) 3	40 (26.3%) 53 (34.9%) 59 (38.8%) 1	0.053
Region^b^ Ontario British Columbia Prairies Quebec Atlantic	170 (55.6%) 41 (13.4%) 41 (13.4%) 30 (9.8%) 24 (7.8%)	85 (55.6%) 21 (13.7%) 20 (13.1%) 15 (9.8%) 12 (7.8%)	85 (55.6%) 20 (13.1%) 21 (13.7%) 15 (9.8%) 12 (7.8%)	1.000
Resides in City with population > 500,000	198 (64.7%)	98 (64.1%)	100 (65.4%)	0.905
Viral suppression of HIV-positive partner Yes No Don’t know Missing	N/A	132 (88.6%) 11 (7.4%) 6 (4.0%) 4	133 (86.9%) 11 (7.2%) 9 (5.9%) 0	0.802
Concurrent relationships Personally involved in concurrent relationships – Yes Partner involved in concurrent relationships – Yes	175 (57.4%) 171 (56.1%)	78 (51.3%) 91 (59.9%)	97 (63.4%) 80 (52.3%)	**0.044** 0.223
Sexual agreement about sex with others – Yes (n = 304)	156 (51.3%)	78 (51.3%)	78 (51.3%)	1.000
Trying to have children with partner^c^ (n = 125)	16 (12.8%)	13 (20.6%)	3 (4.8%)	**0.014**
Relationship satisfaction (range 1–5) *Median (range)*	4.5 (1-5)	4.5 (1-5)	4.5 (1-5)	0.672
Sexual satisfaction Very dissatisfied Somewhat dissatisfied Neither satisfied nor dissatisfied Somewhat satisfied Very satisfied Missing	17 (5.6%) 34 (11.2%) 47 (15.5%) 108 (35.6%) 97 (32.0%) 3	9 (5.9%) 18 (11.8%) 20 (13.2%) 54 (35.5%) 51 (33.6%) 1	8 (5.3%) 16 (10.5%) 27 (17.9%) 54 (35.8%) 46 (30.5%) 2	0.644

^a^Chi-square or Fisher’s exact test for categorical variables, Wilcoxon signed-rank test for ordinal variables, and paired-t test for continuous variables.

^b^While recruitment was conducted in all 10 Canadian provinces and 3 territories, there were no dyads recruited from the territories of the Yukon, Nunavut and the Northwest Territories.

^c^Question only asked of heterosexual partners.

**Table 2:** Relationship characteristics of the current mixed HIV-status couples (N = 153).

Relationship Characteristics	n (%)
Relationship type Male with male Male with female All other	86 (56.2%) 62 (40.5%) 5 (3.3%)
Relationship duration^a^ *Mean (SD)* Less than 1 year 1–2 years 3–5 years 6–14 years 15 years or more	8.5 (8.3%) 17 (11.1%) 25 (16.3%) 35 (22.9%) 42 (27.5%) 34 (22.2%)
HIV in relationship Diagnosis before relationship started Diagnosis and relationship start at same time Diagnosis after relationship started	102 (66.7%) 13 (8.5%) 38 (24.8%)
Duration of HIV in relationship^b^ *Mean (SD)* Less than 1 year 1–2 years 3–5 years 6–14 years 15 or more years	6.7 (6.8%) 25 (16.3%) 26 (17.0%) 36 (23.5%) 42 (27.5%) 24 (15.7%)
Sexual activity (past 3 months) No sexual activity^c^ No penetrative sex^d^ Intercourse – always condoms Intercourse – sometimes condoms Intercourse – never condoms Missing/Don’t know^e^ Not applicable^f^	17 (12.0%) 13 (9.2%) 29 (20.4%) 36 (25.4%) 47 (33.1%) 7 4

^a^Drop-down selection box to record relationship duration ended at “40 or longer,” four dyads reported a relationship lasting 40 years or longer.

^b^Question only asked of HIV-positive partner.

^c^Sexual activity was not reported in the past three months by either partner in the dyad.

^d^In same-sex male relationships, neither partner reported anal insertive or anal receptive intercourse in the past three months. In heterosexual relationships, neither partner reported having sexual intercourse (vaginal or anal) in the past three months.

^e^Neither partner in the dyad responded to questions related to time since sex, frequency of sex, and condom use in the past three months or both partners answered “don’t know” to condom use questions.

^f^Four couples were not asked about participation in penetrative sex due to an inability to participate in penetrative intercourse.

At the time of survey completion, participants reported being in their relationship for a mean of 8.5 years (standard deviation [SD]: 8.3). In a plurality of relationships, condoms were used during intercourse never (33.1%) or sometimes (25.4%) over the past three months. Not all partners reported sexual intercourse with their primary mixed HIV-status partner; 12.0% reported no sexual activity in the relationship and an additional 9.2% reported no penetrative sex.

#### Agreement with U=U.

A total of 293/306 (95.8%) participants responded to the question on agreement with U = U. Approximately half of the participants (53.2%) indicated that they strongly or somewhat agreed with the U = U statement, 15.4% neither agreed nor disagreed, and 31.4% strongly or somewhat disagreed. Individual and relationship characteristics of the 293 participants and 153 relationships are summarized in Tables 3 and 4. A larger proportion of participants agreed with the U = U statement when the HIV-positive partner was virally suppressed compared to when they were not virally suppressed (56.1% versus 32.4%; p = 0.016). The mean age of participants who agreed with the U = U statement was younger compared to those who disagreed with the statement (41.1; SD:12.2 versus 45.2; SD: 10.8; p = 0.006). There was a trend towards a higher proportion of participants from British Columbia (64.1%) or Quebec (72.4%) agreeing with U = U compared to other regions (42.9% to 51.2%; p = 0.083); participants based in the Atlantic provinces were the least likely to agree with the statement (42.9%). Average duration of HIV in the relationship was shorter among dyads who agreed with the U = U statement (mean: 5.0 years; SD: 4.6) compared to those who disagreed (mean: 8.3 years; SD: 7.5) or had opposing views (mean: 6.7 years; SD: 7.4; p = 0.0336). Never using condoms in the past three months was most common among dyads who agreed with the U = U statement (70.2%) compared to those who disagreed (2.1%) or had opposing views (27.7%; p < 0.001).

**Table 3:** Association between demographic characteristics and agreement with the U = U statement^a^ (N = 293 individuals)^b^.

Individual Variables	N	Individual Agreement with U = U statement n (%)	Chi-square p-value
**Viral suppression of HIV positive partner** Supressed Not supressed/Don’t know Missing	255 34 4	143 (56.1%) 11 (32.4%)	**0.016**
**HIV status** HIV-negative HIV-positive	145 148	71 (49.0%) 85 (57.4%)	0.182
Gender * Sexual identity Gay man Heterosexual woman Heterosexual man All else	154 52 53 34	75 (48.7%) 30 (57.7%) 29 (54.7%) 22 (64.7%)	0.317
**Age** (continuous) Agree (mean, SD) Disagree (mean, SD)	156 137	41.4 (12.2) 45.2 (10.8)	**0.006** ^c^
**Racial identity** White Other racial identity Missing	206 83 4	107 (51.9%) 47 (56.6%)	0.554
**Completed education** Less than secondary diploma Secondary diploma or GED Tertiary diploma/degree Missing	29 58 205 1	11 (37.9%) 35 (60.3%) 110 (53.7%)	0.141
**Personal annual income** Less than $20,000 $20,000 - $49,999 $50,000 or more Missing	88 103 98 4	42 (47.7%) 56 (54.4%) 55 (56.1%)	0.486
**Region of Canada** Ontario British Columbia Prairies Quebec Atlantic	163 39 41 29 21	80 (49.1%) 25 (64.1%) 21 (51.2%) 21 (72.4%) 9 (42.9%)	0.083
**Personal concurrent relationships** Yes No Don’t know Missing	124 166 2 1	72 (58.1%) 82 (49.4%) 1 (50.0%)	0.179
**Partner’s concurrent relationships** Yes No Don’t know Missing	100 165 27 1	61 (61.0%) 81 (49.1%) 13 (48.1%)	0.147
**Relationship satisfaction** (range 1–5) Agree (mean, SD) Disagree (mean, SD)	156 137	4.3 (0.7) 4.4 (0.6)	0.666^c^
**Sexual satisfaction** Very dissatisfied Somewhat dissatisfied Neither satisfied nor dissatisfied Somewhat satisfied Very satisfied Missing	16 32 43 106 93 3	8 (50.0%) 10 (31.2%) 24 (55.8%) 59 (55.7%) 54 (58.1%)	0.111

^a^U = U statement: “When a person’s viral load is undetectable they can safely have intercourse with their partner without a condom.”

^b^293 of the 306 participants responded to questions on agreement with U = U. The other 13 respondents responded with “don’t know” or skipped the question.

^c^T-test p-values presented.

**Table 4:** Association between relationship characteristics and agreement with the U = U statement^a^ (N = 153 relationships).

Relationship-level Variables	Number of Dyads	Dyad Agrees with Statement	Dyad Disagrees with Statement	Dyad in Discord	p-value^b^
**Relationship type** Male – Male Male – Female Other	86 61 5	29 (33.7%) 26 (42.6%) 4 (80.0%)	29 (33.7%) 17 (27.9%) 1 (20.0%)	28 (32.6%) 18 (29.5%) 0	0.554
**Relationship duration (years)** N Mean years (SD)	153 8.5 (8.3)	59 6.9 (8.0)	47 10.4 (8.1)	46 8.4 (8.4)	0.095
**Timing of HIV in relationship** HIV before relationship started HIV and relationship at same time HIV after relationship started	101 13 38	39 (38.6%) 5 (38.5%) 15 (39.5%)	36 (35.6%) 2 (15.4%) 9 (23.7%)	26 (25.7%) 6 (46.2%) 14 (36.8%)	0.333
**Duration of HIV in relationship** N Mean years (SD)	153 6.7 (6.8)	59 5.0 (4.6)	47 8.3 (7.5)	46 6.7 (7.4)	**0.034** ^**c**^
**Sexual activity (past 3 months)** Intercourse - never condoms Intercourse - sometimes condoms Intercourse - always condoms No penetration^d^ No sexual activity^e^ Neither partner responded^f^	47 36 29 13 16 7	33 (70.2%) 13 (36.1%) 5 (17.2%) 3 (23.1%) 0 (0%)	1 (2.1%) 10 (27.8%) 16 (55.2%) 7 (63.8%) 10 (62.5%)	13 (27.7%) 13 (36.1%) 8 (27.6%) 3 (23.1%) 6 (37.5%)	**<0.001**
**Concurrent relationships while in relationship** Both monogamous in relationship With another >3 months ago With another < 3 months ago Missing	69 35 46 2	26 (37.7%) 12 (34.3%) 20 (43.5%)	25 (36.2%) 12 (34.3%) 9 (19.6%)	18 (26.1%) 11 (31.4%) 17 (37.0%)	0.375
**Relationship satisfaction – mean score of the two partners** N Mean satisfaction (SD)	153 4.4 (0.5)	59 4.4 (0.5)	47 4.4 (0.4)	46 4.3 (0.6)	0.200

^a^U = U statement: “When a person’s viral load is undetectable they can safely have intercourse with their partner without a condom.”

^b^Chi-square or Fisher’s exact test for categorical variables, ANOVA for continuous variables.

^c^Tukey HSD test for post-hoc differences were run to determine which comparative groups were significantly different: i) Dyad agrees with statement vs. Dyad disagrees with statement: p = **0.026** ii) Dyad agrees with statement vs. Dyad in discord: p = 0.366, iii) Dyad disagrees with statement vs. Dyad in discord p = 0.462.

^d^In same-sex male relationships, neither partner reported anal insertive or anal receptive intercourse in the past three months. In heterosexual relationships, neither partner reported having sexual intercourse (vaginal or anal) in the past three months.

^e^Sexual activity was not reported in the past three months by either partner in the dyad.

^f^Neither partner in the dyad responded to questions related to time since sex, frequency of sex, and condom use in the past three months or both partners answered “don’t know” to condom use questions.

### Response concordance within dyads

Agreement with the U = U statement within dyads exhibited moderate concordance (k = 0.351; S1 Table). In those partnerships where there was discordance, HIV-positive partners trended towards endorsing the statement (p = 0.105; Table 5). Concordance was higher on variables where partners were reporting shared activities such as length of relationship (concordance = 93.5%), involvement in concurrent relationships (k = 0.723), condom use (k = 0.627), and viral suppression of the positive partner (k = 0.558). Concordance was lower on subjective questions such as sexual satisfaction (k = 0.400) and relationship satisfaction (concordance = 13.7%).

**Table 5:** Partners’ concordance and discordance in agreeing or disagreeing with the Undetectable = Untransmittable (U = U) statement^a^ and reporting relationship-level variables (N = 153 dyads).

Dichotomous/Categorical relationship-level variables	Concordance N (%)	Discordance N (%)	Kappa	Kappa p-value	Bias test p-value^b^
Agreement with U = U statement^a^ (2-point scale agree versus disagree/neutral; n = 141)	95 (67.4%)	46 (32.6%)	0.351	<0.0001	0.105
Sexual activity – 3 months (3-category: intercourse; sex, no penetration; no sex; n = 87)^c^	71 (81.6%)	16 (18.4%)	0.644	<0.0001	0.188
Condom use within the last 3 months (3 category: never, sometimes, always; n = 73)^c^	55 (75.3%)	18 (24.7%)	0.627	<0.0001	-^d^
Viral suppression of positive partner (yes/no; n = 149)	135 (90.6%)	14 (9.4%)	0.558	<0.0001	0.789
Sexual agreement (yes/no; n = 151)	112 (74.2%)	39 (25.8%)	0.483	<0.0001	1.000
Sexual Satisfaction (5-point Likert scale; n = 150)	60 (40.0%)	90 (60.0%)	0.400	<0.0001	0.894
Concurrent relationships (yes/no; n = 152)	126 (82.9%)	26 (17.1%)	0.723	<0.0001	0.228
Continuous relationship-level variables	Concordance N (%)	Bland-Altman 95% Limits of Agreement	–	–	Bland-Altman Bias (95% CI) with respect to HIV-positive partner
Length of relationship (years; n = 153)	143 (93.5%)	(−2.6, 2.2)	–	–	−0.24 (−0.04, −0.43)
Relationship Satisfaction (1–100 scale; n = 153)	21 (13.7%)	(−36.0, 37.4)	–	–	0.6 (−2.6, 3.6)

^a^U = U statement: “When a person’s viral load is undetectable they can safely have intercourse with their partner without a condom.”

^b^McNemar or McNemar-Bowker test for categorical variables, Wilcoxon signed-rank test for ordinal variables.

^c^There was missing data on this variable due to a nuance in survey design. If a participant did not indicate when they first and last had sex (how many years/months ago) or indicated that they did not know the answer, these questions were skipped by the online survey. For partners who were together longer, there was a greater probability that one of them was less certain of the timing of their first sexual contact and selected “don’t know” as their response, and thus all sexual activity questions were skipped. However, generally one of the partners was able to answer the question and provide sexual activity data.

^d^Test could not be run due to zero counts in two of the nine cells.

^e^Condoms for HIV-prevention statement: “Condoms will protect a person from getting HIV through sex with their partner when the partner’s viral load is detectable.”

^f^HIV management strategies statement: “I feel confident that our HIV management strategies can keep me [my partner] from becoming HIV positive.”

#### Association between dyad-level belief in U=U and condomless sex.

Table 6 shows the crude (n = 142 dyads) and adjusted (n = 140 dyads) associations between dyad-level agreement with U = U and always having sexual intercourse without a condom in the past three months. In unadjusted analyses, dyads who agreed with the U = U statement and dyads who had opposing views had 37.64 (95% CI: 9.29, 345.15) and 7.22 (95%. CI: 1.23, 75.68) times higher odds of reporting condomless sex compared to dyads who disagreed with the U = U statement. Unadjusted, dyads with a virally suppressed HIV-positive partner reported 5.85 (95% CI: 1.78, 29.97) higher odds of condomless sex compared to dyads with HIV-positive partners with unsupressed or unknown viral suppression status. Unadjusted, a longer relationship duration was associated with lower odds of condomless sex (OR: 0.91; 95% CI: 0.85, 0.97).

**Table 6:** Association between agreement with the U = U statement^a^ and always having condomless sex in the past 3 months.

Characteristic	Unadjusted Odds Ratio (N = 142)		Adjusted Odds Ratio^b^ (N = 140)	
	OR (95% CI)	p-value	aOR (95% CI)	p-value
Dyad agreement with U = U statement^a,c^ Dyad disagrees/ambivalent Dyad agrees Dyad has opposing views	1.00 37.64 (9.29, 345.15) 7.22 (1.23, 75.68)	**<0.001** **0.028**	1.00 0.76 (0.01, 16.49) 2.94 (0.19, 47.77)	0.866 0.419
Viral suppression of HIV-positive partner ^c^ Not virally supressed/don’t know Supressed	1.00 5.85 (1.78, 29.97)	**0.002**	1.00 0.15 (0, 3.3)	0.231
Interaction term: agreement with U = U * Viral suppression^d^ Dyad disagrees/ambivalent * Not virally suppressed/don’t know Dyad agrees * Virally suppressed Dyad opposed * Virally suppressed	–	–	1.00 122.39 (2.78, 36361.95) 4.89 (0.12, 1052.68)	**0.013** 0.416
Relationship type Male – male Male – female Male – other	1.00 1.09 (0.53, 2.20) 2.09 (0.16, 26.72)	0.817 0.535	1.00 1.41 (0.55, 3.74) 2.26 (0.06, 868.65)	0.476 0.736
Age of HIV-positive partner (years, centered)	0.90 (0.76, 1.05)	0.174	1.19 (0.93, 1.55)	0.166
Age difference between partners (years)	0.97 (0.81, 1.17)	0.772	0.93 (0.73, 1.18)	0.558
Duration of HIV in relationship (years)	0.91 (0.85, 0.97)	**0.002**	0.89 (0.81, 0.97)	**0.009**

^a^U = U statement: “When a person’s viral load is undetectable they can safely have intercourse with their partner without a condom.”

^b^The adjusted odds ratio was further adjusted for duration of HIV in the relationship (in years), relationship type, age of the HIV-positive partner, and age difference between partners.

^c^Due to the presence of an interaction term, the adjusted odds ratios for the main effects (Dyad agreement with U = U statement and Viral suppression of HIV-positive partner) should be interpreted in combination with the interaction term and not in isolation. Refer to S2 Table for linear combination of main effects and interaction.

**Fig 1:**
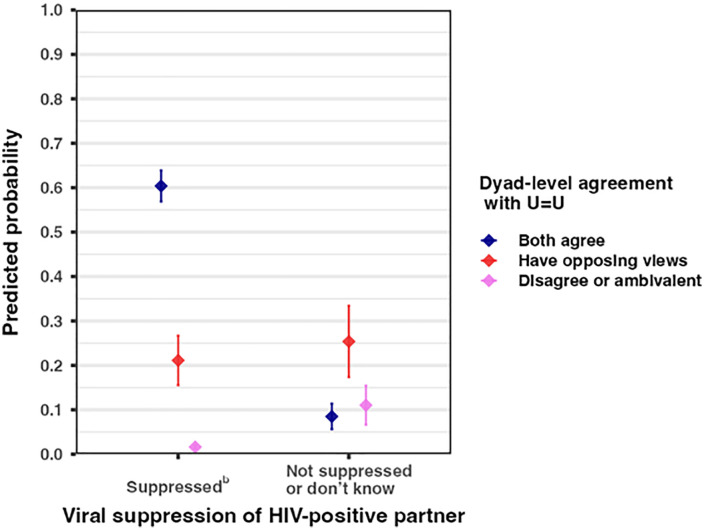
Predicted probability of always having condomless sex in the past 3 months by dyad-level agreement with the U = U statement^a^ and HIV-positive partner’s viral suppression status. (N = 140 relationship dyads). ^a^U = U statement: “When a person’s viral load is undetectable they can safely have intercourse with their partner without a condom.” ^b^Both partners reported that the HIV-positive partner was virally suppressed.

Multivariable logistic regression revealed a significant interaction of dyad-level agreement with U = U and viral suppression of the HIV-positive partner on condomless sex. The linear combination of main effects and interaction are presented in S2 Table. Compared to dyads who disagreed with U = U and were not confident that the HIV-positive partner was virally suppressed, dyads who agreed with U = U and were confident had 14.38 (95% CI: 2.30, 89.72) times higher odds of engaging in condomless sex (S2 Table). The predicted probability of condomless sex in the three months prior to survey completion among dyads who agreed with U = U and were confident of viral suppression was 0.60 (95% CI: 0.57, 0.64; Fig 1; S2 Table). The likelihood of condomless sex was low (predicted probability: 0.08; 95% CI: 0.06, 0.11) when the HIV-positive partner was not virally suppressed, even if both partners agreed with U = U. When only one partner (either the HIV-positive or HIV-negative partner) agreed with U = U, the likelihood of condomless sex remained low with (predicted probability: 0.21; 95% CI: 0.16, 0.27) or without (predicted probability: 0.25; 95% CI: 0.12, 0.33) confidence in viral suppression.

## Discussion

Our findings suggested that agreement with U = U in mixed HIV-status relationships was dependent on age, duration of HIV in the relationship, and province/region of residence, and was associated with never using a condom in the past three months. Further, our study confirms the key role that viral suppression plays in modifying the association between dyad-level agreement with U = U and condomless sex. To the best of our knowledge, this was one of few studies that examined agreement with U = U among both partners in mixed HIV-status relationships and the first study to quantify the impact of belief in U = U on condomless sex among people involved in mixed HIV-status relationships.

The proportion of participants who agreed with U = U in our sample (approximately half) was similar to the proportion of sexual minority men who agreed with U = U in a different study that took place in the United States (53.2%) [53], but smaller than the proportion of gay and bisexual men in Australia who agreed with U = U (67.3%) [54]. In our study, proportions of participants who resided in British Columbia or Quebec who agreed with U = U tended to be higher compared to participants who resided in other regions including Ontario, the Prairies, and the Atlantic provinces. These differences may be due to regional discrepancies in U = U messaging, resources allocated to HIV prevention education, or HIV-related stigma. For example, a previous study found that while HIV-related stigma was less common, undetectable viral load was more common in Vancouver compared to other regions in Canada including Alberta, a prairie province [55]. Our findings were similar to prior studies showing that younger age was associated with U = U agreement [45,46]. In our sample, participants who reported low condom use and were in relationships where the duration of HIV in the relationship was shorter were more likely to agree with U = U, suggesting that U = U messaging was able to reach, and was trusted by, these populations. On the other hand, a smaller proportion of participants who were older and involved in mixed HIV-status relationships that were impacted by HIV over a longer period agreed with U = U. Our findings demonstrated the need for more effective public health messaging regarding U = U to improve understanding and agreement with U = U among wider populations.

Our study identified an association between agreement with U = U and condom use that was modified by the HIV-positive partner’s viral suppression status. Specifically, we found that people involved in primary mixed HIV-status relationships had the highest odds of condomless sex if both partners agreed with U = U and the HIV-positive partner in the relationship was virally suppressed. Participants who were in a relationship where the HIV-positive partner was not virally suppressed had lower odds of condomless sex, regardless of whether one or both partners agreed with the U = U statement. This finding built on a previous Canadian study that demonstrated that the likelihood of condom use increased with a perceived a risk of being diagnosed with a sexually transmitted infection in the next six months [56]. Our findings regarding the associations between U = U agreement, viral suppression, and condom use may reflect our participants’ sophisticated understanding of viral suppression as a crucial component of U = U and their ability to clearly distinguish situations with a higher versus lower risk of HIV transmission. As per existing evidence and U = U, condom use when the HIV-positive partner was not virally suppressed helped to prevent the transmission of HIV [57] while condomless sex when the HIV-positive partner is virally suppressed does not present a risk of HIV transmission [18–21]. Effective public health messaging about U = U, understanding which situations create a higher risk of HIV transmission, and the use of appropriate protective measures (e.g., condom use) when needed may also help to increase sexual pleasure while reducing HIV-related stigma, HIV transmission, and the overcriminalization of HIV non-disclosure [25–27].

Notably, our findings show that the occurrence of condomless sex may be dependent on whether both partners agreed with U = U and whether both partners were confident that the HIV-positive partner in the relationship was virally suppressed. If only one partner agreed with U = U and/or were confident in the viral suppression status of the HIV-positive partner, then condomless sex was less likely to occur. This finding emphasizes the importance of involving both mixed HIV-status partners in research studies as the views of both partners in a relationship play a critical role in their health decisions and sexual behaviors [47]. When asking both partners in a relationship to respond to the same questions in a research study, it is unavoidable that there will be concordant and discordant responses between the two partners. Like previous studies, our study observed higher concordance in responses between partners for factual questions (e.g., length of relationship, condom use, viral suppression of the HIV-positive partner, involvement in concurrent relationships) [58] and lower concordance on subjective questions about beliefs and decision-making (e.g., agreement with U = U, sexual satisfaction, relationship satisfaction) [59]. Lower concordance can be explained, in part, as an artefact of the kappa statistic used to measure concordance. Kappa values can be low when responses are highly skewed (e.g., very common or very rare) for both partners, even if there were few discordant responses between partners [60]. As the kappa statistic may underestimate the concordance in responses between partners, it has become common for researchers to accept low kappa values in studies [61].

Our study had several limitations. First, we focused on people in primary mixed HIV-status relationships so findings may not be applicable to casual relationships. Due to the focus on involving both partners in primary mixed HIV-status relationships, our study had a small sample size despite being a national study, which may limit the generalizability of our study findings. The small sample size also led to wide confidence intervals in some associations, including the association between U = U agreement and condomless sex. Second, our study may have been susceptible to volunteer bias given the absence of a pre-defined sampling frame (e.g., existing registry). Most study participants self-identified as gay men, White, and were involved in longer-term mixed HIV-status relationships. Previous studies have found that people who self-identify as men who have sex with men may have higher awareness and agreement with U = U compared to other populations (e.g., Black heterosexually active adults) [40–43]. Additionally, people who self-identify as men or White tend to be less willing to use condoms compared to women and other ethnic/racial minorities [16,36,37,56]. Despite efforts to control for potential confounding, study findings may be biased away from the null hypothesis (i.e., no association between U = U agreement and condomless sex), leading to an overestimation of the association between agreement with U = U and condomless sex. As previous research has also shown that males [62] and people who self-identify as White [63] have higher odds of reporting viral suppression, this may further exacerbate these biases. Third, although individuals in polyamorous relationships were eligible, each index participant was paired with only one primary mixed HIV-status partner for dyadic analysis, potentially introducing selection bias. Participants were not asked about polyamory so sensitivity analyses could not be conducted. Future studies should examine the association between U = U agreement and condomless sex in this population. Fourth, skip patterns in the survey design generated some missing data in participant-level responses to condom use questions. Specifically, if a participant did not indicate when they first and last had sex or indicated “don’t know,” sexual behavior questions were skipped by the online survey. To mitigate this issue, given the high concordance in responses between partners who both answered, when only one partner responded, we imputed their response as the dyad-level variable. Fifth, social desirability bias could have led to underreporting of sexual behavior and overreporting of condom use. When both partners responded to these questions but their responses were discordant, we assigned the dyad the most conservative response. This method may have resulted in overestimation of the frequency of condomless sex. Finally, the cross-sectional design did not account for changes in HIV care and U = U agreement and awareness over time and limited the causal interpretation of any associations identified in our study.

## Conclusions

These findings suggest that agreement with the U = U concept and viral suppression of the HIV-positive partner within mixed HIV-status relationships played an important role in their decisions to have condomless sex. Our findings suggested a need for clearer U = U messaging to advance the understanding of, and agreement with U = U. Future representative studies among sexual identity and racialized minority sub-populations are needed to better understand how mixed HIV-status relationships receive and apply U = U messaging, with a focus on partner age and the duration of HIV within relationships. All partners in relationships should be included in future studies on these topics to ensure that findings reflect the diverse perspectives of people engaged in mixed HIV-status relationships.

## Supporting information

S1 Table Details on concordance and discordance for U = U statement,^a^ viral suppression,^b^ and condom use,^c^ between HIV-positive and HIV-negative partners in a dyad. ^a^ U = U statement: “When a person’s viral load is undetectable they can safely have intercourse with their partner without a condom.” Don’t know and missing responses are omitted from the significance tests. ^b^ Viral suppression at <50 coplies/mL. Missing responses are omitted from the significance tests. ^c^ Condom use always, sometimes, never. No intercourse, no sexual contact, and missing are grouped together and omitted from the significance tests. ^d^ Significance tests exclude the “don’t know and missing” category, N = 141. ^e^ Significance tests exclude the “missing” category, N = 149. ^f^ The Kappa significance test excludes the “No intercourse, no sexual activity, and missing” category. N = 73. McNemar-Bowker bias test could not be run due to zero cell counts.
(PDF)

S2 Table Adjusted odds ratio (linear combination of main effects and interaction) and predicted probability of always having condomless sex in the past 3 months by agreement with the U = U statement^a^ and viral suppression of HIV-positive partner. **(N = 140).** ^a^ U = U statement: “When a person’s viral load is undetectable they can safely have intercourse with their partner without a condom.” ^b^ At least one partner responded “unsuppressed” or “don’t know.” ^c^ Due to the use of an interaction term to examine the role of viral suppression of the HIV-positive partner as a potential effect modifier, this is the referent group for all adjusted odds ratios presented in the table.
(PDF)

S3 File Data access request agreement document. (DOC)
