# Supplementary material for: The impact of Undetectable=Untransmittable and viral suppression on condomless sex among mixed HIV-status couples in Canada
Source: PLoS One. 2025 Oct 9;20(10):e0332926. doi: 10.1371/journal.pone.0332926 (PMC12510518; doi:10.1371/journal.pone.0332926)
Supplement: S1 Table — Don’t know and missing responses are omitted from the significance tests. b Viral suppression at <50 coplies/mL. Missing responses are omitted from the significance tests. c Condom use always, sometimes, never. No intercourse, no sexual contact, and missing are grouped together and omitted from the significance tests. d Significance tests exclude the “don’t know and missing” category, N = 141. e Significance tests exclude the “missing” category, N = 149. f The Kappa significance test excludes the “No intercourse, no sexual activity, and missing” category. N = 73. McNemar-Bowker bias test could not be run due to zero cell counts. (PDF) [file pone.0332926.s001.pdf]

**S1 Table. Details on concordance and discordance for U=U statement,[a] viral suppression,[b] and condom use,[c] between HIV-positive and HIV-negative partners in a dyad.**

| | | | **HIV negative partner's response** | | | | Kappa p-value | McNemar-Bowker Bias test p-value |
|---|---|---|---|---|---|---|---|---|
| | | | U=U statement agreement | | | | | |
| | | | Agree | Disagree | Don't know/ missing | | | |
| **HIV positive partner's response** | U=U statement agreement | Agree | 44 | 17 | 2 | | 0.351 [d] P<0.0001 | 0.105 |
| | | Disagree | 29 | 51 | 5 | | | |
| | | Don't know/ missing | 1 | 3 | 1 | | | |
| | | | | | | | | |
| | | | | | | | | |
| | | | Positive Partner's suppression | | | | | |
| | | | Suppressed | Unsuppressed/ Don't Know | Missing | | | |
| | Positive partner's suppression | Suppressed | 124 | 8 | 0 | | 0.558 [e] P<0.0001 | 0.789 |
| | | Unsuppressed/ don't know | 6 | 11 | 0 | | | |
| | | missing | 3 | 1 | 0 | | | |
| | | | | | | | | |
| | | | | | | | | |
| | | | Condom use | | | | | |
| | | | Never | Sometimes | Always | No sex/ missing | | |
| | Condom use | Never | 22 | 6 | 0 | 6 | 0.627 P<0.0001 | -- [f] |
| | | Sometimes | 4 | 17 | 3 | 5 | | |
| | | Always | 1 | 4 | 16 | 6 | | |
| | | No sex/ missing | 7 | 9 | 8 | 39 | | |

[a] U=U statement: "When a person's viral load is undetectable they can safely have intercourse with their partner without a condom." Don't know and missing responses are omitted from the significance tests.
[b] Viral suppression at <50 copies/mL. Missing responses are omitted from the significance tests.
[c] Condom use always, sometimes, never. No intercourse, no sexual contact, and missing are grouped together and omitted from the significance tests.
[d] Significance tests exclude the "don't know and missing" category, N=141
[e] Significance tests exclude the "missing" category, N=149
[f] The Kappa significance test excludes the "No intercourse, no sexual activity, and missing" category. N=73. McNemar-Bowker bias test could not be run due to Zero cell counts.
