# Supplementary material for: The impact of Undetectable=Untransmittable and viral suppression on condomless sex among mixed HIV-status couples in Canada
Source: PLoS One. 2025 Oct 9;20(10):e0332926. doi: 10.1371/journal.pone.0332926 (PMC12510518; doi:10.1371/journal.pone.0332926)
Supplement: S2 Table — (N = 140). a U = U statement: “When a person’s viral load is undetectable they can safely have intercourse with their partner without a condom.” b At least one partner responded “unsuppressed” or “don’t know.” c Due to the use of an interaction term to examine the role of viral suppression of the HIV-positive partner as a potential effect modifier, this is the referent group for all adjusted odds ratios presented in the table. (PDF) [file pone.0332926.s002.pdf]

S2 Table. Adjusted odds ratio (linear combination of main effects and interaction) and predicted probability of always having condomless sex in the past 3 months by agreement with the U=U statement[a] and viral suppression of HIV-positive partner. (N=140)

**Viral Suppression of HIV-Positive Partner**	**Agreement with U=U Statement[a]**	**Adjusted Odds Ratio**	**Predicted Probability (95% CI)**
1) Unsupressed[b]	1) Dyad disagrees/ambivalent	1[c]	0.11 (0.07, 0.15)
	2) Dyad agrees	0.76 (0.01, 16.49)	0.08 (0.06, 0.11)
	3) Dyad has opposing views	2.94 (0.19, 47.77)	0.25 (0.17, 0.33)
2) Suppressed	1) Dyad disagrees/ambivalent	0.15 (0, 3.30)	0.02 (0.01, 0.02)
	2) Dyad agrees	14.38 (2.30, 89.72)	0.60 (0.57, 0.64)
	3) Dyad has opposing views	2.22 (0.28, 17.97)	0.21 (0.16, 0.27)

[a] U=U statement: “When a person’s viral load is undetectable they can safely have intercourse with their partner without a condom.”
[b] At least one partner responded "unsuppressed" or “don’t know.”
[c] Due to the use of an interaction term to examine the role of viral suppression of the HIV-positive partner as a potential effect modifier, this referent group for all adjusted odds ratios presented in the table.
