# Supplementary material for: The impact of Undetectable=Untransmittable and viral suppression on condomless sex among mixed HIV-status couples in Canada
Source: PLoS One. 2025 Oct 9;20(10):e0332926. doi: 10.1371/journal.pone.0332926 (PMC12510518; doi:10.1371/journal.pone.0332926)
Supplement: S3 File — (DOC) [file pone.0332926.s003.doc]

**
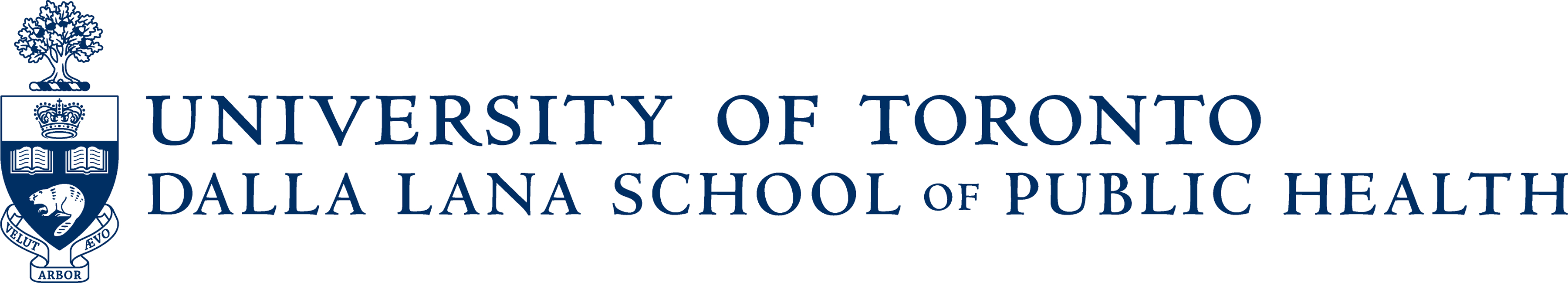
**

# Positive Plus One: A study of relationships

# where one partner is HIV positive and the other is HIV negative

**Data Access Request Agreement for Study Investigators and Collaborators**

# I ____________________________________________ request access to the following data (e.g., variables, transcripts, etc.) please specify:

# For the purpose of:

In requesting these data, I declare that I have no conflict of interest in using these raw data, and have no client or patient relationship with any participants.

To access these data, I am required to obtain approval by an official Research Ethics Board for the jurisdiction of the requesting researcher, as well as from the University of Toronto Research Ethics Board.

# I agree to regard all data related to *Positive Plus One: A study of relationships where one partner is HIV positive and the other is HIV negative* (the Project) as strictly confidential and as the sole property of the University of Toronto.

I will protect all information to ensure full confidentiality. This obligation applies to information in any form (e.g., written, electronic or oral). I agree that the terms outlined in this agreement will remain in force even if I cease to have an association with the Project.

I agree to respect the following rules regarding the treatment all study data:

- I will only access confidential information that I need to know to analyse data, prepare research findings or to meet other responsibilities with the Project.
- I will not discuss or disclose any confidential information pertaining to this study, whether written or verbal, with anyone other than research team members.
- I will maintain the anonymity of study participants.
- I agree to keep all confidential material in a locked place and understand the need to ensure that confidential material are not left lying around for others to see.
- I agree to keep all digital (electronic files) on a password protected computer and will not share my password with anyone.
- I will accurately analyse and report all data.
- I will not falsify or fabricate data or change any responses.
- I will not use any data in a manner not approved by the research team.
- I will immediately report the fact that confidential information in my possession has been stolen or lost as well as any other violations of the above rules to the *Positive Plus One* Study Principal Investigator without threat of penalty for doing so.
- All study data and analyses will be returned to the Study Office for keeping, and deleted from working computers, once manuscripts have been accepted.

I have read this agreement and will comply with the conditions outlined herein.

Signature of individual making data request Date

Signature Date

Dr. Liviana Calzavara

Positive Plus One, Principal Investigator
